# Association of controlling nutritional status with fecal incontinence and fecal incontinence severity index in individuals with spinal cord injury: mediating roles of interleukin-6 and uric acid

**DOI:** 10.3389/fnut.2026.1842981

**Published:** 2026-08-17

**Authors:** Ying Yu, Hao Xiong, Di Wang, Yin Wang, Sijian Lin, Xiang Chen, Meilan Zhu, Liu Yang

**Affiliations:** 1 Department of Rehabilitation Medicine, The Second Affiliated Hospital, Jiangxi Medical College, Nanchang University, Nanchang, China; 2 Department of Orthopaedics, Affiliated Hospital of Jiangxi University of Traditional Chinese Medicine, Jiangxi University of Traditional Chinese Medicine, Nanchang, China; 3 Department of General Practice, The Second Affiliated Hospital, Jiangxi Medical College, Nanchang University, Nanchang, China

**Keywords:** controlling nutritional status, fecal incontinence, fecal incontinence severity index, mediation effect, spinal cord injury

## Abstract

**Background:**

Fecal incontinence (FI) is common after spinal cord injury (SCI), but modifiable risk factors are unclear. The association between malnutrition and FI in patients with SCI remains unclear.

**Methods:**

This cross-sectional study included 2,256 SCI patients. Nutritional status was assessed using the CONUT score (albumin, cholesterol, lymphocytes). FI was self-reported, and the fecal incontinence severity index (FISI) was used to quantify severity. Logistic and linear regressions tested CONUT associations with FI and FISI. Restricted cubic splines examined the dose–response. Exploratory mediation analyses assessed IL-6 and uric acid (UA). The predictive performance of CONUT was evaluated using AUC, NRI, and IDI.

**Results:**

Among 2,256 patients, 267 (11.8%) reported FI. After fully adjusting for covariates, higher CONUT was linked to increased FI odds (OR per 1-unit increase, 1.27; 95%CI, 1.09–1.67) and higher FISI (*β* = 0.51; 95%CI, 0.32–0.74) in a near-linear dose–response manner. The findings were consistent across subgroups and sensitivity analyses. CONUT showed moderate predictive ability for FI (AUC = 0.788; 95%CI, 0.759–0.818), outperforming the geriatric nutritional risk index (AUC = 0.632) and prognostic nutritional index (AUC = 0.626). Adding CONUT improved reclassification (NRI = 0.19; 95%CI, 0.11–0.30; IDI = 0.12; 95%CI, 0.06–0.20). In exploratory mediation analyses, IL-6 accounted for 16.37 and 18.21% of the total association with FI and FISI, respectively; UA accounted for 14.89 and 10.11%, respectively.

**Conclusion:**

CONUT was positively and nearly linear-dose-responsively associated with FI odds and severity, with IL-6 and UA partially accounting for these associations. Among three nutritional indices examined, CONUT demonstrated the strongest association with FI severity. However, these findings reflect relative performance among nutritional markers rather than established clinical predictive utility.

## 1 Introduction

Spinal cord injury (SCI) is defined as organic damage to the spinal cord tissue resulting from trauma (e.g., fracture-dislocation) or pathological conditions (e.g., neoplasm, inflammation, ischemia) (1). This injury leads to temporary or permanent loss of motor, sensory, sphincter (bladder and bowel), and autonomic function below the level of injury. Based on the preservation of sacral function, SCI is classified as either complete or incomplete (2). Current epidemiological data indicate that the global annual incidence of SCI is approximately 13 to 15 cases per million population, corresponding to 250,000 to 500,000 new cases each year (3). The worldwide prevalence is estimated at 236 to 1,000 cases per million population, with a total of 20 to 30 million individuals living with SCI (4). Mortality of SCI follows a bimodal distribution: prehospital mortality in the acute phase is as high as 20 to 40%, whereas in-hospital mortality has decreased to 5 to 10% in high-income countries but remains substantially higher in low- and middle-income countries (5). These findings underscore the substantial and growing disease burden of SCI worldwide. SCI may cause several complications, such as pneumonia, urinary tract complications (e.g., renal failure), pressure injuries, autonomic dysreflexia, and deep vein thrombosis (6–10). Additionally, fecal incontinence and constipation are frequently observed gastrointestinal complications (11). These complications frequently coexist, interact, and perpetuate a vicious cycle that both shortens life expectancy and profoundly impairs quality of life. Consequently, the prevention and management of complications have become central, long-term priorities in the comprehensive care of individuals with SCI.

Fecal incontinence (FI), a complication of spinal cord injury, refers to the involuntary passage of solid or liquid stool, or the inability to control bowel movements under normal social and hygienic conditions, and this symptom is persistent (typically lasting at least three months) (12). Current research indicates that risk factors influencing the occurrence of fecal incontinence include neurogenic factors (e.g., spinal cord injury, Parkinson’s disease, stroke), anatomical and structural factors (e.g., anal sphincter injury and laxity of pelvic floor support structures), bowel function and stool consistency factors (e.g., chronic diarrhea, chronic constipation), cognitive and behavioral factors (e.g., Alzheimer’s disease, dementia, limited mobility), and demographic and lifestyle factors (e.g., age, gender, obesity, medication use) (13–16). However, the studies on the risk factors of FI in individuals with SCI remain limited. In recent years, the importance of nutrition status in delaying the onset and progression of the disease and improving treatment outcomes and prognosis has been demonstrated (17). Therefore, the nutritional status of the vulnerable population has been widely attended to. Growing evidence has supported that malnutrition is associated with the risk of FI and SCI (18, 19). Currently, the association between malnutrition and FI in individuals with SCI remains unclear.

Malnutrition is a clinical condition characterized by measurable adverse alterations in body composition, physiological function, and clinical outcomes, arising from inadequate, excessive, or imbalanced intake of energy, protein, and other nutrients, including vitamins and minerals (20). The Controlling Nutritional Status (CONUT) score is a screening tool used to assess nutritional status quickly and objectively. It is calculated using three routine blood parameters: serum albumin, total cholesterol, and peripheral blood lymphocyte count (21). Compared to commonly used clinical nutritional indicators (such as BMI, serum albumin, and prealbumin), CONUT offers the following advantages: the calculated scores are entirely objective, with no assessor bias; there are no additional costs, as it is based on routine blood tests; and it provides a comprehensive assessment of protein reserves, energy reserves, and immune status through multidimensional indicators (22). Therefore, CONUT is an efficient, cost-effective, and objective nutritional screening tool suitable for the early identification of malnutrition, the early warning of complication risks, and the guidance of individualized nutritional interventions (23). However, it should be noted that the CONUT score has not been specifically validated in the SCI population. Therefore, the association of CONUT with the risk of FI in individuals with SCI remains unclear.

To address the above-mentioned research gaps, we conducted a cross-sectional study to explore the association between CONUT and FI in patients with SCI. This study could validate CONUT as a practical tool for identifying high-risk individuals who may benefit from targeted nutritional support, thereby offering a potential pathway to reduce FI prevalence and improve overall prognosis in individuals with SCI.

## 2 Methods

### 2.1 Study design

A retrospective cross-sectional analysis was conducted to investigate the relationship between the CONUT and FI among individuals diagnosed with SCI. Data were derived from health examination records spanning January 1, 2018, to January 1, 2025, obtained from the Center of Rehabilitation Medicine in The Second Affiliated Hospital of Jiangxi Medical College. The research protocol received ethical approval from the institutional review boards of the participating hospital (O-MRER [2026] No. 020). All participants provided written informed consent before enrollment, in accordance with the ethical standards outlined in the Declaration of Helsinki.

**Figure 1:**
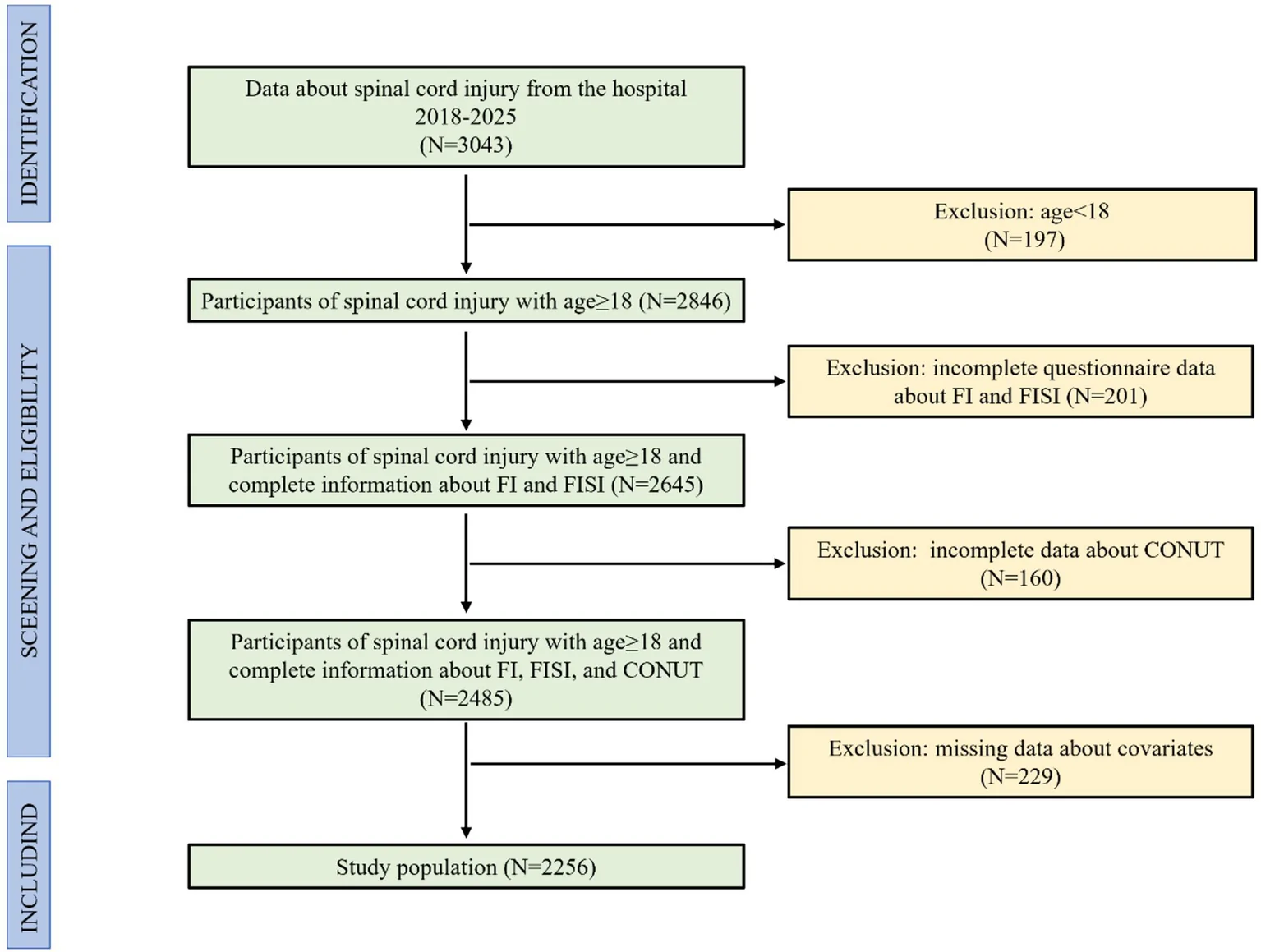
Flow chart of participants in inclusion and exclusion criteria.

In this study, of the initial 3,043 participants, 197 were excluded for being younger than 18 years. Among the remaining individuals, further exclusions were made due to missing data: 210 for FI and FISI, 160 for CONUT, and 229 for covariates. After these sequential exclusions, the final analytic cohort comprised 2,256 patients of SCI aged 18 years or older with complete information on all study variables. A detailed flowchart of the participant selection process is provided in Figure 1.

### 2.2 Calculation of CONUT

The CONUT score is calculated from three laboratory measures: serum albumin, total cholesterol, and total lymphocyte count (24). Albumin levels are scored as 0 for ≥3.5 g/dL, 2 for 3.0 to 3.49 g/dL, 4 for 2.5 to 2.99 g/dL, and 6 for <2.5 g/dL. Total cholesterol is scored as 0 for ≥180 mg/dL, 1 for 140 to 179 mg/dL, 2 for 100 to 139 mg/dL, and 3 for <100 mg/dL. Lymphocyte count is scored as 0 for ≥1,600/mm^3^, 1 for 1,200 to 1,599/mm^3^, 2 for 800 to 1,199/mm^3^, and 3 for <800/mm^3^ (25). The total score ranges from 0 to 12, with higher scores indicating worse nutritional status. Scores of 0 to 1 indicate normal nutrition, 2 to 4 mild malnutrition, 5 to 8 moderate malnutrition, and 9 to 12 severe malnutrition (26).

### 2.3 Definition of SCI, FI, and FISI

According to the international standards for neurological classification of spinal cord injury (ISNCSCI, 2019 revision), the diagnostic classification of spinal cord injury is based on a standardized neurological examination. Completeness of injury is determined by assessing sacral (S4-S5) function, including sensory testing (light touch, pinprick, and deep anal pressure) and voluntary anal sphincter contraction. Sensory levels are derived from testing light touch and pinprick at 28 key points, and motor levels from manual muscle testing of 10 key muscles (27). The neurological level of injury is defined as the lowest spinal segment with intact bilateral sensory and motor function. Injuries are classified using the ASIA impairment scale (AIS), ranging from A (complete, no sacral sensory or motor function preserved) to E (normal) (28). In complete injuries, the zone of partial preservation is documented as the lowest segment below the neurological level with preserved sensory or motor function. FI was assessed using the bowel health questionnaire (BHQ). Participants identified their predominant stool consistency via the Bristol Stool Form Scale (BSFS) cards and reported the type and frequency of accidental bowel leakage over the preceding 30 days (29). FI was defined as any involuntary passage of mucus, liquid, or solid stool during this period. Severity of accidental bowel leakage was quantified using the fecal incontinence severity index (FISI) questionnaire (30). This instrument evaluated four distinct categories of leakage (gas, mucus, liquid, and solid stool) across six frequency gradations, from never to twice or more daily. Each category-frequency combination carries a specific weight (ranging from 0 to 19), and the aggregate FISI score is derived by summing these weighted values. Higher composite scores reflect greater severity of incontinence (31).

### 2.4 Assessment of covariates

Our study included the following sociodemographic, lifestyle, and clinical biomarker covariates. Sociodemographic and lifestyle covariates included: age (years); sex (male, female); educational attainment (<high school, high school, ≥college); body mass index (<18.5, 18.5–24.9, 25.0–29.9, ≥30.0 kg/m^2^); tobacco and alcohol use (never, former, current). Hypertension was defined as mean systolic blood pressure ≥130 mm Hg or diastolic blood pressure ≥80 mm Hg, physician diagnosis, or antihypertensive medication use (32). Diabetes was defined as fasting glucose ≥126 mg/dL, hemoglobin A1c ≥ 6.5%, clinician diagnosis, or glucose-lowering medication use (33). Cardiovascular disease included coronary heart disease, cerebrovascular disease, peripheral arterial disease, aortic atherosclerosis and aneurysms, hypertensive heart disease, heart failure, rheumatic heart disease, and congenital heart anomalies. Each disease condition was treated as binary (yes or no) (34). total cholesterol (TC), triglyceride (TG), low-density lipoprotein cholesterol (LDL), high-density lipoprotein cholesterol (HDL), glycated hemoglobin A1c (HbA1c), fasting glucose, C-reactive protein (CRP), uric acid (UA), albumin (ALB), aspartate aminotransferase (AST), alanine aminotransferase (ALT), gamma-glutamyl transferase (GGT), and lactate dehydrogenase (LDH), and interleukin-6 (IL-6) were included. Key indicators of SCI progression were also included: time since injury (<1 year, 1–5 years, >5–10 years, >10 years); injury level (cervical, thoracic, lumbosacral); and ASIA Impairment Scale grade (A, no sensory or motor function preserved; B, sensory but no motor function preserved; C, motor function preserved with >50% of key muscles having grade <3; D, motor function preserved with ≥50% of key muscles having grade ≥3) (35, 36).

### 2.5 Statistical analysis

Baseline characteristics were compared using *t*-tests (continuous variables) and *χ*^2^ tests (categorical variables). The CONUT score was analyzed as a continuous variable and a categorical variable in quartiles. Multivariable logistic and linear regression models assessed the associations of CONUT with FI and FISI. Nonlinear relationships were examined using restricted cubic splines (RCS). Subgroup analyses stratified by age, sex, education, BMI, alcohol use, tobacco use, physical activity, hypertension, and diabetes evaluated the consistency of findings. Predictive performance of CONUT for FI was evaluated using receiver operating characteristic curves (ROC) to calculate sensitivity, specificity, accuracy, positive predictive value (PPV), and negative predictive value (NPV). Comparisons with the geriatric nutritional risk index (GNRI) and prognostic nutritional index (PNI) were performed, with incremental predictive value assessed using net reclassification improvement (NRI) and integrated discrimination improvement (IDI). Sensitivity analyses included: (1) multiple imputation using chained equations (MICE) with 20 imputed datasets; (2) adjustment for medications (e.g., antibiotics and opioids); (3) exclusion of individuals with vascular, neoplastic, infectious, or gastrointestinal conditions; (4) additional adjustment for inflammatory factors (CRP, TNF-*α*, and IL-1*β*); and (5) additional adjustment for bowel management strategies (laxative use, enema use, digital evacuation, antidiarrheal medications, and structured bowel management programs). Mediation analyses were used to explore the roles of IL-6 and UA. Due to the cross-sectional design, the causal effects of IL-6 and UA on the association between CONUT and FI and FISI cannot be established. The mediation effects reported represent statistical associations rather than causal pathways or proven biological mechanisms, which should be interpreted as exploratory and hypothesis-generating. All analyses were conducted using R version 4.3.3, with statistical significance defined as two-sided *p* < 0.05.

## 3 Results

### 3.1 Baseline characteristics of the participants

Table 1 summarizes baseline characteristics of the 2,256 participants who met the inclusion criteria. Of these, 50.8% (*n* = 1,147) were male, 38.6% (*n* = 870) had completed college or higher, and 267 (11.8%) reported FI. Among participants with SCI, those with FI differed significantly from those without across most covariates (*p* < 0.05). Additionally, individuals with FI had higher CONUT scores, IL-6 levels, and uric acid levels than those without FI.

**Table 1:** Baseline characteristics of the participants.

Variables	Total (*n* = 2,256), *n* (%) or mean (se)	FI (*n* = 267), *n* (%) or mean (se)	Non-FI (*n* = 1,989), *n* (%) or mean (se)	*p*-value
Age	50.25 (5.34)	54.57 (6.05)	47.38 (5.03)	<0.001
Gender				
Female	1,109 (49.16)	130 (48.69)	979 (49.22)	<0.001
Male	1,147 (50.84)	137 (51.31)	1,010 (50.78)	
Educational levels				
Less than high-school	593 (26.29)	83 (31.09)	510 (25.64)	<0.001
High school	793 (35.15)	80 (29.96)	713 (35.85)	
College or above	870 (38.56)	104 (38.95)	766 (38.51)	
BMI				
Underweight	404 (17.90)	70 (26.22)	334(16.79)	<0.001
Normal weight	882 (39.10)	44 (16.48)	838(42.13)	
Overweight	684 (30.32)	72 (26.96)	612(30.77)	
Obesity	286 (12.68)	81 (30.34)	205(10.31)	
Drinking status				
Never	592 (26.24)	58 (21.72)	534 (26.85)	<0.001
Former	736 (32.62)	89 (33.33)	647 (32.53)	
Current	928 (41.14)	120 (44.95)	808 (40.62)	
Smoking status				
Never	657 (29.12)	53 (19.85)	604 (30.37)	<0.001
Former	643 (28.50)	76 (28.46)	567 (28.51)	
Current	956 (42.38)	138 (51.69)	818 (41.12)	
Physical levels				
Vigorous level	730 (32.36)	73 (27.34)	657 (33.03)	<0.001
Middle level	705 (31.25)	82 (30.71)	623 (31.32)	
Low level	821 (36.39)	112 (41.95)	709 (35.65)	
Hypertension				
Yes	954 (42.29)	89 (33.33)	865 (43.49)	<0.001
No	1,302 (57.71)	178 (66.67)	1,124 (56.51)	
Diabetes				
Yes	874 (38.74)	78 (29.21)	796 (40.02)	<0.001
No	1,382 (61.25)	189 (70.79)	1,193 (59.98)	
Cardiovascular disease				
Yes	871 (38.61)	69 (25.84)	802 (40.32)	<0.001
No	1,385 (61.39)	198 (74.16)	1,187 (59.68)	
FISI	6.86 (2.69)	16.3 (3.43)	3.13 (1.26)	<0.001
CONUT	2.45 (0.18)	2.80 (0.29)	2.00 (0.10)	<0.001
TC (mg/dL)	216.05 (6.02)	220.17 (6.76)	214.45 (5.39)	<0.001
TG (mg/dL)	123.26 (5.26)	126.44 (6.53)	120.16 (4.47)	<0.001
HDL (mg/dL)	36.04 (4.35)	34.86 (5.21)	39.45 (3.90)	<0.001
HbA1c (%)	5.05 (1.03)	6.13 (1.25)	4.56 (0.76)	0.009
Fast glucose (mg/dL)	90.12 (6.15)	93.56 (7.23)	88.25 (5.39)	<0.001
CRP (mg/L)	3.25 (1.00)	4.05 (1.15)	2.80 (0.80)	<0.001
UA (mg/dL)	6.23 (1.64)	6.96 (2.35)	5.85 (1.20)	0.042
AST (U/L)	19.85 (3.05)	21.96 (3.35)	18.50 (2.80)	<0.001
ALB (g/L)	44.76 (3.82)	46.65 (4.04)	43.50 (3.40)	<0.001
ALT (U/L)	27.05 (4.05)	29.20 (4.90)	24.80 (3.25)	<0.001
GGT (U/L)	34.65 (3.54)	37.46 (4.32)	33.24 (3.16)	<0.001
LDH (U/L)	135.37 (5.27)	138.55 (6.24)	133.25 (4.46)	<0.001
IL-6 (pg/mL)	3.45 (0.47)	3.89 (0.62)	3.04 (0.39)	<0.001
Time since injury (years)				
<1	520 (23.05)	64 (23.97)	456 (22.93)	0.002
1–5	598 (26.51)	66 (24.72)	532 (26.75)	
5–10	712 (31.56)	89 (33.33)	623 (31.32)	
>10	426 (18.88)	48 (17.98)	378 (19.00)	
Level of SCI				
Cervical	998 (44.24)	130 (48.69)	868 (43.65)	<0.001
Thoracic	752 (33.33)	78 (29.21)	674 (33.88)	
Lumbosacral	506 (22.43)	59 (20.97)	447 (22.47)	
ASIA impairment scale				
A (Complete)	937 (41.53)	125 (46.82)	812 (40.82)	0.001
B (Sensory incomplete)	447 (19.81)	42 (15.72)	405 (20.36)	
C (Motor incomplete)	470 (20.83)	52 (19.48)	418 (21.02)	
D (Motor incomplete)	402 (17.83)	48 (17.98)	354 (17.80)	

BMI: body mass index, TC: total cholesterol, TG: triglyceride, LDL: low-density lipoprotein cholesterol, HDL: high-density lipoprotein cholesterol, HbA1c: glycated hemoglobin A1c, CRP: C-reactive protein, UA: uric acid, ALB: albumin, ALT: alanine aminotransferase, AST: aspartate aminotransferase, GGT: gamma-glutamyl transferase, LDH: lactate dehydrogenase. Categorical data are summarized as percentages, with group comparisons evaluated using *χ*^2^ tests. Continuous variables are expressed as means (standard errors), and differences between groups were assessed via unpaired *t*-tests. A two-sided *p* value < 0.05 indicated statistical significance.

### 3.2 Association between CONUT, FI, and FISI in individuals with SCI

Table 2 revealed positive relationships between the CONUT and both FI and FISI among SCI patients. Each one-unit increment in CONUT was associated with 45% (Model I: odds ratio [OR], 1.45; 95% CI, 1.23–1.82), 38% (Model II: OR, 1.38; 95% CI, 1.18–1.76), and 27% (Model III: OR, 1.27; 95% CI, 1.09–1.67) higher odds of FI.

**Table 2:** Association between CONUT, FI, and FISI in individuals with SCI.

Models	OR (95%CI)	*p*-value	*β* (95%CI)	*p*-value
Model I				
Continuous	1.45 (1.23, 1.82)	<0.001	0.73 (0.45, 0.99)	<0.001
Q1	Reference		Reference	
Q2	1.06 (0.95, 1.23)	0.122	0.13 (−0.12, 0.35)	0.114
Q3	1.37 (1.15, 1.64)	<0.001	0.52 (0.35, 0.84)	<0.001
Q4	1.63 (1.38, 1.94)	<0.001	0.85 (0.63, 1.22)	<0.001
*p* for trend	0.001		<0.001	
Model II				
Continuous	1.38 (1.18, 1.76)	<0.001	0.62 (0.38, 0.88)	<0.001
Q1	Reference		Reference	
Q2	1.01 (0.90, 1.15)	0.103	0.08 (−0.20, 0.27)	0.167
Q3	1.24 (1.07, 1.53)	0.002	0.45 (0.27, 0.76)	<0.001
Q4	1.52 (1.29, 1.85)	<0.001	0.76 (0.52, 1.01)	<0.001
*p* for trend	0.004		0.001	
Model III				
Continuous	1.27 (1.09, 1.67)	<0.001	0.51 (0.32, 0.74)	<0.001
Q1	Reference		Reference	
Q2	0.95 (0.83, 1.08)	0.232	−0.02 (−0.31, 0.25)	0.258
Q3	1.15 (1.01, 1.33)	0.043	0.28 (0.16, 0.58)	<0.001
Q4	1.45 (1.18, 1.72)	<0.001	0.67 (0.43, 0.89)	<0.001
*p* for trend	0.008		0.002	

Q1: quartile 1; Q2: quartile 2; Q3: quartile 3; Q4: quartile 4. OR: odds ratio. Model I is a crude model. Model II adjusted for age, gender, and educational levels. Model III adjusted for all covariates, except for UA and IL-6. *p* < 0.05 was regarded as statistically significant.

**Figure 2:**
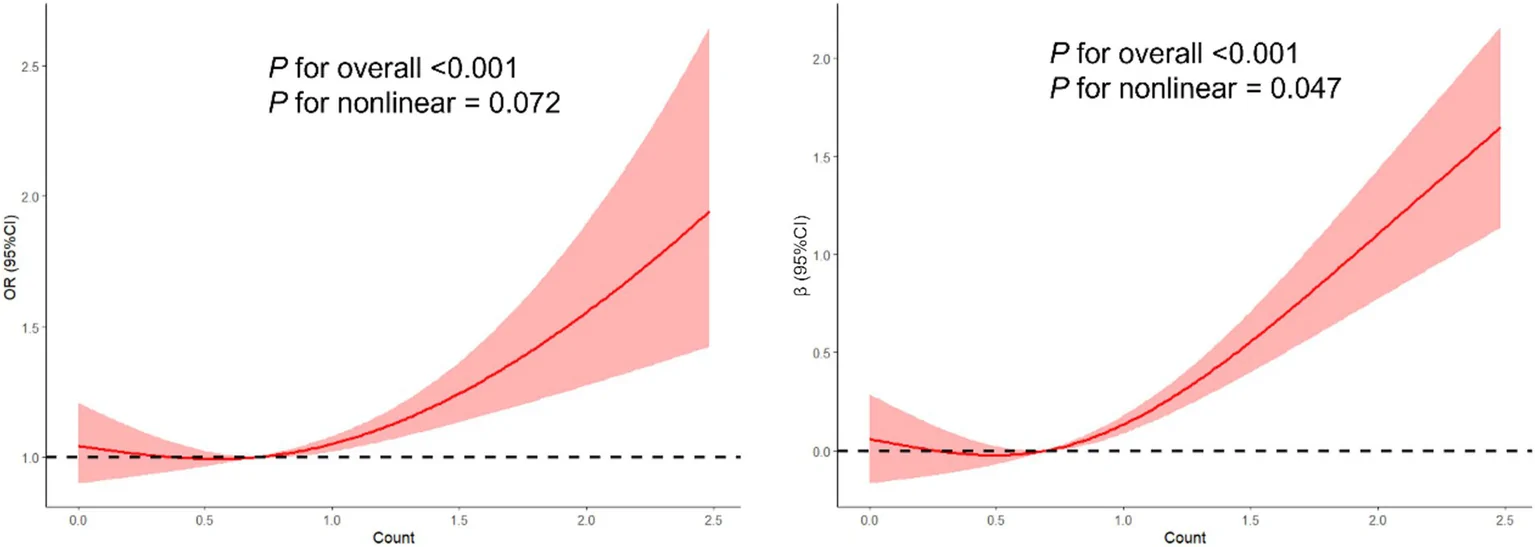
The nearly linear associations of the CONUT with FI and its severity (FISI) using RCS models adjusted for all covariates. The solid lines represent the estimated odds ratios for FI and regression coefficients for FISI, with shaded bands indicating the corresponding 95% confidence intervals, based on a 3-knot spline specification. The plots illustrate a progressive increase in both FI risk and FISI scores as CONUT increases.

When examined by quartiles, participants in the highest quartile (Q4) exhibited a 63% (Model I: OR, 1.63; 95% CI, 1.38–1.94), 52% (Model II: OR, 1.52; 95% CI, 1.29–1.85), and 45% (Model III: OR, 1.45; 95% CI, 1.18–1.72) increase in FI risk relative to those in the lowest quartile (Q1). Similar findings were also observed for incontinence severity: each unit increase in CONUT corresponded to FISI increases of 0.73 (Model I: *β* = 0.73; 95% CI, 0.45 to 0.99), 0.62 (Model II: *β* = 0.62; 95% CI, 0.38 to 0.88), and 0.51 (Model III: *β* = 0.51; 95% CI, 0.32 to 0.74). Comparing the lowest quartile group, the highest quartile group demonstrated FISI increases of 0.85 (Model I: *β* = 0.85; 95% CI, 0.63 to 1.22), 0.76 (Model II: *β* = 0.76; 95% CI, 0.52 to 1.01), and 0.67 (Model III: *β* = 0.67; 95% CI, 0.43 to 0.89). Trend analysis confirmed gradually increasing relationships with the increase of CONUT level across all models (all *p* for trend < 0.05). Restricted cubic splines further substantiated nearly linear associations for both outcomes (FI: *p* for nonlinear = 0.074, overall *p* < 0.001; FISI: *p* for nonlinear = 0.047, overall *p* < 0.001), as depicted in Figure 2.

### 3.3 Subgroup analyses

In this study, subgroup analyses were performed to examine the consistency of the association between the CONUT and both FI and its severity (FISI) among patients with SCI. Stratification variables included age, sex, educational attainment, BMI status, alcohol and tobacco use, physical activity levels, hypertension, diabetes, and cardiovascular disease. As presented in Table 3 and Supplementary Table S1, CONUT was positively associated with FI and FISI across various subgroups (e.g., participants aged <60 years: OR, 1.18; 95% CI, 1.08–1.33; *β* = 0.54; 95% CI, 0.47 to 0.64; those aged ≥60 years: OR, 1.37; 95% CI, 1.15–1.60; *β* = 0.46; 95% CI, 0.38 to 0.57). No significant effect modification was detected for any of the stratification variables (all *p* for interaction >0.05).

**Table 3:** Association between CONUT and FI in patients with SCI stratified by various subgroups.

Subgroups	OR (95%CI)	*p*-value	*p* for interaction
Age			
<60	1.18 (1.08, 1.33)	<0.001	0.302
≥60	1.37 (1.15, 1.60)	<0.001	
Gender			
Female	1.22 (1.14, 1.43)	<0.001	0.358
Male	1.35 (1.23, 1.58)	<0.001	
Educational levels			
Less than High-school	1.23 (1.17, 1.35)	<0.001	0.415
High school	1.17 (1.08, 1.38)	0.002	
College or above	1.36 (1.14, 1.49)	<0.001	
BMI			
Underweight	1.07 (0.97, 1.24)	0.075	0.306
Normal weight	1.15 (1.06, 1.31)	0.009	
Overweight	1.23 (1.14, 1.42)	<0.001	
Obesity	1.41 (1.27, 1.68)	<0.001	
Drinking status			
Never	1.05 (0.96, 1.21)	0.068	0.229
Former	1.18 (1.09, 1.31)	<0.001	
Current	1.30 (1.14, 1.53)	<0.001	
Smoking status			
Never	1.10 (1.02, 1.24)	0.033	0.387
Former	1.14 (1.06, 1.28)	0.005	
Current	1.36 (1.25, 1.54)	<0.001	
Physical levels			
Vigorous	1.16 (1.08, 1.34)	<0.001	0.264
Middle	1.07 (0.98, 1.20)	0.065	
low	1.38 (1.30, 1.54)	<0.001	
Hypertension			
Yes	1.40 (1.29, 1.62)	<0.001	0.398
No	1.12 (1.03, 1.34)	0.023	
Diabetes			
Yes	1.34 (1.18, 1.56)	<0.001	0.419
No	1.19 (1.09, 1.30)	<0.001	
Cardiovascular disease			
Yes	1.31 (1.16, 1.52)	<0.001	0.397
No	1.25 (1.05, 1.37)	0.004	

BMI: body mass index, OR: odds ratio. All covariates were adjusted in the model, except for UA and IL-6. *p* < 0.05 was regarded as statistically significant.

### 3.4 Increased predictive performance of CONUT on the FI in individuals with SCI

**Figure 3:**
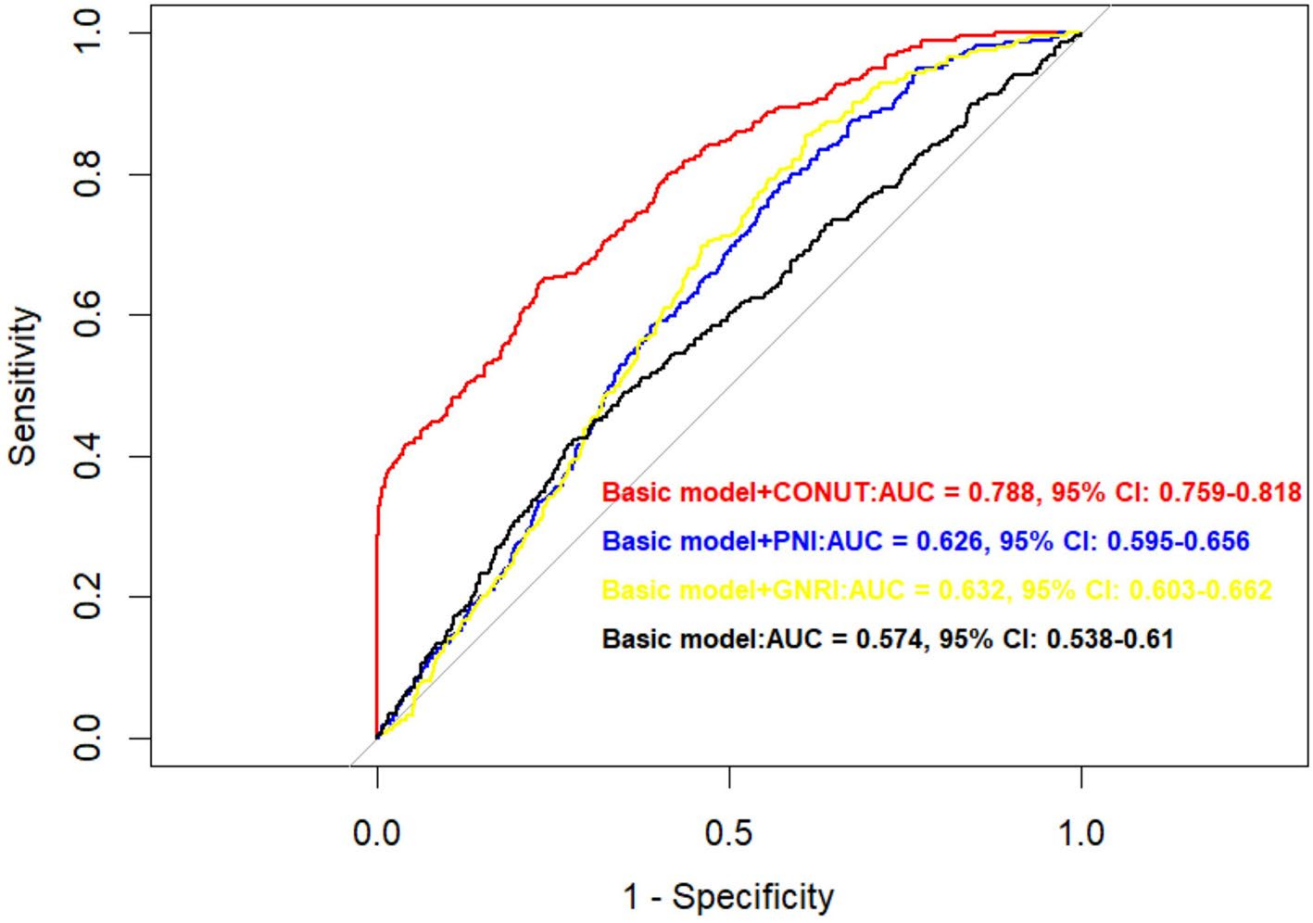
The predictive effect of some malnutrition indicators (PNI, GNRI, and CONUT) on the FI in individuals with SCI. The basic model means all covariates, except for IL-6 and UA, were adjusted.

The predictive value of the CONUT for FI was evaluated using ROC curves, as shown in Figure 3 and Supplementary Table S2. Based on the basic model, CONUT demonstrated moderate discriminative ability, with an area under the curve of 0.788 (95% CI, 0.759–0.818), sensitivity of 0.738, and specificity of 0.701. Compared with other malnutrition indices, CONUT exhibited superior predictive performance (*p* < 0.05; Supplementary Table S3). Incremental predictive capacity was further assessed using NRI and IDI. As presented in Table 4, the addition of CONUT significantly improved FI risk classification (NRI, 0.19; 95% CI, 0.11–0.30; *p* < 0.001) and discrimination (IDI, 0.12; 95% CI, 0.06–0.20; *p* < 0.001), indicating its enhanced utility for identifying FI in SCI patients.

**Table 4:** Discriminative performance of the diagnosis of FI in individuals with SCI.

Models	NRI	*p*-value	IDI	*p*-value
Basic model	Reference		Reference	
Basic model + GNRI	0.08 (0.03, 0.14)	0.005	0.03 (0.01, 0.08)	0.042
Basic model + PNI	0.07 (0.02, 0.13)	0.036	0.02 (−0.01, 0.06)	0.063
Basic model + CONUT	0.19 (0.11, 0.30)	<0.001	0.12 (0.06, 0.20)	<0.001

NRI: net reclassification improvement, IDI: integrated discrimination improvement, GNRI: geriatric nutritional risk index, PNI: prognostic nutritional index. Basic model included all covariates, except for IL-6 and UA. *p* < 0.05 was regarded as statistically significant.

### 3.5 The mediation effects of the UA and IL-6

**Figure 4:**
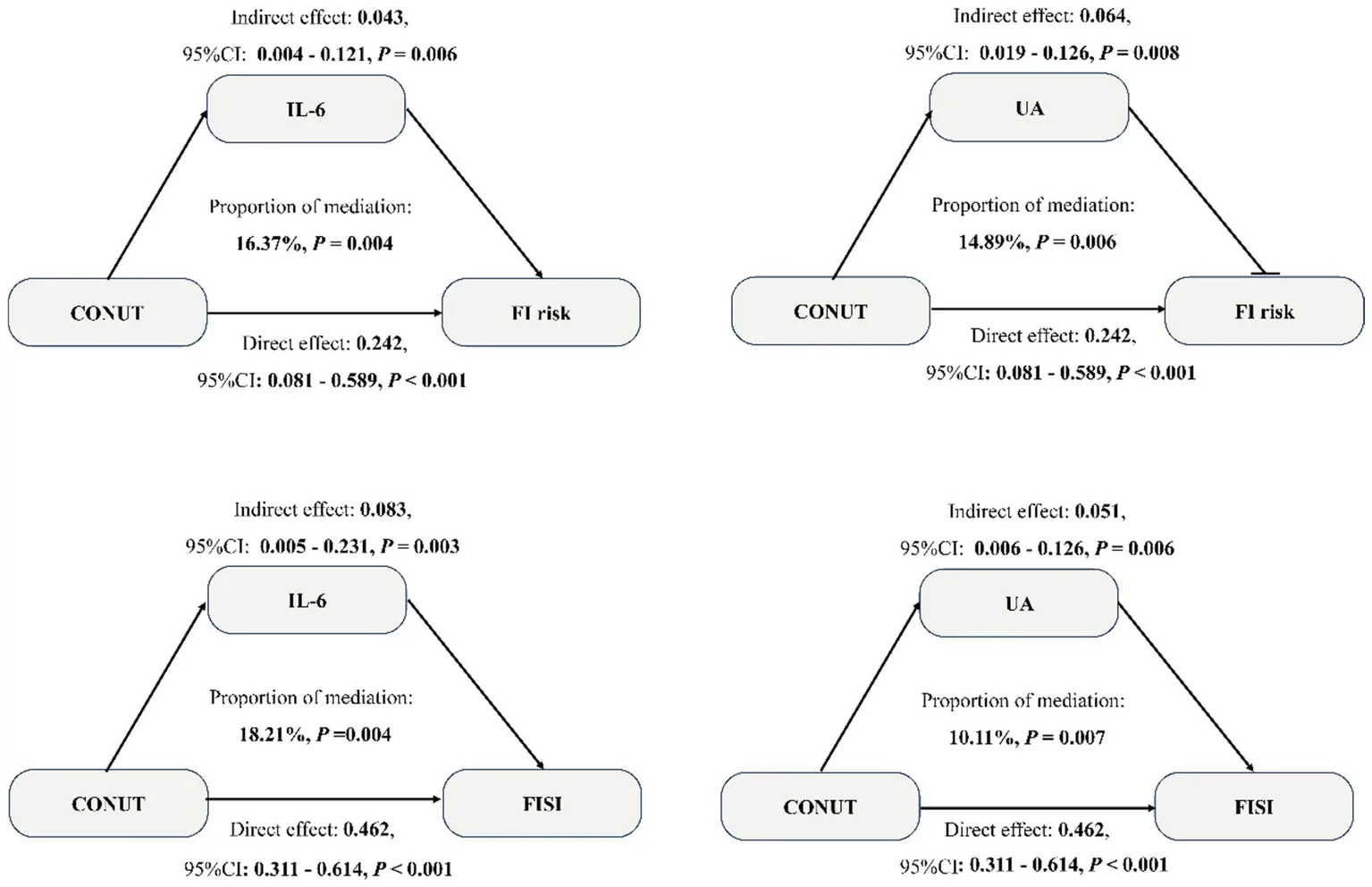
Exploratory mediation showing the statistical associations between CONUT, inflammatory markers (IL-6, UA), FI, and FISI. All paths represent cross-sectional statistical associations based on single-time-point data, not responding to the causal effect. Both biomarkers emerged as significant mediators, accounting for a considerable portion of the total association.

An exploratory mediation analysis examined the potential mediating roles of UA and IL-6 in the relationship between the CONUT and both FI and its severity (FISI) in patients with SCI. As shown in Supplementary Table S4, after full covariate adjustment, IL-6 was positively associated with FI (OR = 1.52; 95% CI, 1.41–1.65; *p* < 0.001) and FISI (*β* = 0.65; 95% CI, 0.50–0.81; *p* < 0.001). Similarly, UA demonstrated positive associations with FI (OR = 1.65; 95% CI, 1.50–1.89; *p* < 0.001) and FISI (*β* = 0.76; 95% CI, 0.64–0.89; *p* < 0.001). In addition, both biomarkers were also positively associated with CONUT (Supplementary Table S5). After full covariate adjustment, IL-6 was positively related to CONUT for FI (*β* = 0.54; 95% CI, 0.45 to 0.67; *p* < 0.001) and FISI (*β* = 0.68; 95% CI, 0.55 to 0.83; *p* < 0.001), as was UA (FI: *β* = 0.65; 95% CI, 0.55 to 0.78; *p* < 0.001; FISI: *β* = 0.79; 95% CI, 0.66 to 0.93; *p* < 0.001). Mediation analysis further revealed that IL-6 accounted for 16.37 and 18.21% of the total effect of CONUT on FI and FISI, respectively, while UA accounted for 14.89 and 10.11%, respectively (Figure 4). Given the cross-sectional design, the analyses were exploratory and could not establish temporal precedence or causal mediation. Our results should be interpreted as hypothesis-generating only.

### 3.6 Sensitivity analyses

Multiple sensitivity analyses were performed to assess the robustness of the associations between the CONUT and both FI and its severity (FISI) in SCI patients. First, multiple imputation was employed to address potential bias from missing data; the positive associations remained essentially unchanged after imputation (Supplementary Table S6). Second, to evaluate the influence of comorbid conditions, we reanalysed the data after excluding individuals with a history of vascular, neoplastic, infectious, or gastrointestinal conditions. The positive relationships persisted in our analyses (Supplementary Table S7). Third, given that certain medications (e.g., antibiotics and opioids) may promote bowel incontinence, we additionally adjusted for these agents in the regression models; the positive associations again remained robust (Supplementary Table S8). Because CONUT scores may reflect inflammatory status in addition to malnutrition, we reperformed the multivariable logistic regression and linear models to assess the robustness of this association by additionally adjusting for inflammatory factors. Our results showed that the positive association remained consistent with the primary findings (Table S9). In addition, several SCI-specific factors may substantially influence bowel function, including bowel management programs, laxative use, enema use, digital evacuation, and antidiarrheal medications. Therefore, to minimise these effects on this association, we reassessed the robustness of this association after additionally adjusting for these confounding factors. Our result showed that the positive association remained stable (Supplementary Table S10).

## 4 Discussion

In this retrospective cross-sectional study, we examined the association between the CONUT score and FI and FISI in patients with SCI. CONUT was positively associated with both outcomes in a near-linear dose–response manner. The association was consistent across subgroups and in sensitivity analyses. CONUT demonstrated moderate predictive capacity for FI, outperforming other nutritional indices such as the PNI and GNRI, with improvements in discrimination reflected by positive NRI and IDI values. Mediation analyses suggested that IL-6, a marker of inflammation, and UA, a marker of oxidative stress, partially accounted for these associations.

Bowel dysfunction, particularly FI, represents a prevalent complication among individuals with SCI, yet its modifiable risk factors remain incompletely defined. Emerging evidence suggests that malnutrition may play an important role in the development of post-SCI complications (37). For instance, a study by Huang et al. demonstrated that preoperative malnutrition, assessed using the GNRI, was independently associated with increased postoperative complications in patients undergoing spinal surgery, including those with traumatic SCI (38). Similarly, Esli Samaniego et al. reported that low serum albumin, a key component of nutritional assessment, was a significant predictor of adverse outcomes, including infectious complications, in patients with acute SCI (39). It is noted that CONUT has not been specifically validated in the SCI population, and the specific relationship between malnutrition and FI in the SCI population has not been well characterized. The CONUT score, a validated and widely used nutritional screening tool, integrates serum albumin, total cholesterol, and lymphocyte count to objectively assess nutritional status (40). Despite its established utility in predicting outcomes across various clinical contexts, its association with FI in patients with SCI remains unexplored. Leveraging real-world clinical data, the present study sought to investigate this association and explore its potential mechanistic pathways, including the mediating roles of inflammatory and oxidative stress markers.

In this study, 11.8% of patients with SCI reported FI, a proportion comparable to that reported in other similar studies (e.g., a single-center cross-sectional study from India reported a prevalence of approximately 13%) (41). This consistency supports the representativeness of the study population relative to clinical settings. Patients with SCI and FI had higher CONUT scores, IL-6 levels, and UA levels than those without FI. These findings suggest that malnutrition may represent a modifiable risk factor for FI in this population, highlighting the potential for nutritional optimization as an intervention. In addition, the observed elevations in IL-6 and UA point to underlying inflammatory and oxidative stress pathways that may contribute to the pathogenesis or severity of FI in patients with SCI. The finding that the association between the CONUT score and both FI and FISI in patients with SCI was positive and exhibited a dose–response relationship indicates that poorer nutritional status is associated with progressively worse bowel dysfunction in a graded manner. This dose–response pattern strengthens the credibility of the association, as it suggests a biologically plausible relationship rather than a spurious finding. Clinically, this implies that even incremental worsening in nutritional status may correspond to measurable declines in bowel function, underscoring the importance of early nutritional assessment and intervention. Patients with milder nutritional impairment may benefit from timely nutritional support to prevent progression to more severe bowel dysfunction, whereas those with more pronounced malnutrition may require intensive nutritional rehabilitation to mitigate the associated burden of FI and improve overall quality of life. CONUT score has not been specifically validated in the SCI population. In addition, individuals with SCI exhibit unique metabolic, inflammatory, and body composition characteristics that may fundamentally affect CONUT score interpretation. SCI is associated with metabolic dysregulation (e.g., altered lipid metabolism, insulin resistance) that may influence cholesterol levels independently of nutritional status (42). Additionally, SCI is characterized by chronic systemic inflammation, driven by factors such as muscle denervation, pressure ulcers, recurrent infections, and gut microbiome alterations, which can suppress albumin and lymphocyte counts through inflammatory pathways rather than nutritional deficiency alone (43). Furthermore, SCI-induced changes in body composition, including skeletal muscle loss and increased adiposity, may confound the relationship between nutritional biomarkers and clinical outcomes (44). These features suggest that established CONUT cut-offs, developed primarily in general medical and oncology populations, may not be directly generalizable to individuals with SCI. Therefore, our findings in the cross-sectional design regarding its association with FI in the SCI population should be interpreted as exploratory and hypothesis-generating. Future studies should formally validate the utility of CONUT in SCI using large, population-based prospective cohorts.

In addition, this positive association remains stable in various subgroups, and this association was not modified by factors such as age, sex, educational attainment, BMI levels, lifestyle behaviors, or comorbid conditions, which indicates that the relationship between malnutrition and bowel dysfunction in patients with SCI is robust and generalizable, to a certain extent. From a clinical perspective, this consistency suggests that the association is not driven by or limited to specific patient populations but rather reflects a fundamental relationship that holds across diverse clinical and demographic strata. This strengthens the evidence that malnutrition may serve as a universal risk factor for FI in patients with SCI, independent of other patient characteristics, and supports the broad applicability of nutritional assessment and intervention strategies across the entire SCI population. However, this single-center, cross-sectional study was conducted in a Chinese population; therefore, the generalizability of the findings may be limited. Future international multicenter studies are warranted to confirm these results in broader populations.

In exploring the predictive capacity of CONUT on FI of patients with SCI, it demonstrated moderate predictive capacity, yet outperformed other nutritional indices, including PNI and GNRI. Furthermore, the addition of CONUT significantly improved the discriminatory ability for identifying patients at risk for FI, as reflected by positive NRI and IDI values. These findings have important clinical implications. The superior predictive performance of CONUT compared with alternative nutritional tools suggests that its composite assessment of serum albumin, total cholesterol, and lymphocyte count may capture a broader or more relevant spectrum of nutritional derangements contributing to bowel dysfunction in SCI. However, our finding that CONUT outperformed GNRI and PNI in this cohort should be interpreted with caution. The superiority of CONUT in this specific sample does not establish that CONUT has robust independent value for FI prediction in clinical practice. Multiple factors, including cohort characteristics, outcome definitions, and analytical approaches, may influence the comparative performance of nutritional indices, and our findings may not generalize to other SCI populations or settings. Therefore, we do not advocate for the immediate adoption of CONUT into routine clinical practice based solely on these cross-sectional data.

Some studies have demonstrated that inflammation and oxidative stress play important roles in the pathogenesis and progression of both SCI and FI (45–47). For instance, Bolen et al. demonstrated that elevated proinflammatory cytokines, including CRP, were associated with worse neurological outcomes and bowel dysfunction in patients with acute SCI (48). Similarly, Vajdi et al. reported that oxidative stress markers, such as GGT, were independently linked to the severity of secondary injury cascades and functional recovery after SCI (49). Given these established relationships, we hypothesized that inflammation and oxidative stress might be associated with nutritional status, as measured by the CONUT score, and FI in patients with SCI. Our mediation analyses supported this hypothesis, demonstrating that IL-6, a marker of inflammation, and uric acid, a marker of oxidative stress, partially accounted for the association between CONUT and FI in patients with SCI. These findings suggest that IL-6 and UA may be involved in the association between CONUT and FI. However, the exposure (CONUT score) and the proposed mediator (IL-6) may conceptually overlap. Albumin and lymphocyte count, two of the three components of the CONUT score, are acute-phase reactants that are down-regulated during systemic inflammation. Thus, higher CONUT scores may reflect heightened inflammatory activity rather than malnutrition alone. This overlap is particularly relevant to our mediation analyses, because IL-6, as the proposed mediator, is itself a key inflammatory cytokine. Consequently, the statistical association between CONUT and IL-6 may be partially attributable to shared inflammatory pathways rather than to a causal nutritional-to-inflammatory sequence. This raises the possibility that the proportion of the CONUT-FI association statistically accounted for by IL-6 in our exploratory models may be inflated. We therefore caution against interpreting these findings as evidence of a distinct mediating pathway and emphasize that our results are hypothesis-generating rather than confirmatory. The fact that most of the total association remained unexplained (approximately 80%) indicates that other unmeasured pathways, such as gut microbiota alterations or autonomic dysfunction, likely also contribute (50, 51). These findings have several clinical implications. First, they highlight the potential value of targeting inflammation and oxidative stress as therapeutic strategies to mitigate FI in this population. Second, monitoring IL-6 and UA levels may help identify patients with SCI at heightened risk for FI, enabling earlier and more personalized interventions. Collectively, these findings support a multidimensional approach to managing FI in patients with SCI that addresses not only neurogenic bowel mechanisms but also systemic nutritional, inflammatory, and oxidative stress derangements.

The cross-sectional design of this study offers several advantages in examining the association between CONUT and FI among patients with SCI. First, it enabled the inclusion of a relatively large sample size, enhancing statistical power to detect significant associations while adjusting for multiple confounders. Second, the use of standardized, routinely collected laboratory data, including serum albumin, total cholesterol, and lymphocyte count, ensured objective and reliable CONUT measurement, minimizing information bias. Third, the comprehensive assessment of FI using validated instruments (FISI and BHQ) allowed for precise characterization of incontinence severity. Additionally, several sensitivity analyses strengthen the robustness of the findings. These results provide foundational epidemiological evidence supporting the role of nutritional status in gastrointestinal tract dysfunction, generating hypotheses for future prospective and mechanistic investigations.

Several limitations of this study should be acknowledged. First, the cross-sectional design precludes any causal inference regarding the direction or mechanism of the observed associations. The statistical mediation effects are exploratory and hypothesis-generating and do not establish temporal order. Prospective cohort studies with repeated measurements and experimental studies are needed to establish temporality and test causal hypotheses. Second, the direction of association may be reversed. FI-related complications, including bowel dysfunction, reduced mobility, and swallowing difficulties, may lead to dietary restriction, reduced intake, and subsequent nutritional abnormalities reflected by higher CONUT scores. Our cross-sectional data cannot distinguish the direction of the association, and a bidirectional relationship is also possible. Third, given that two components of the CONUT score are inflammation-sensitive, the inability to differentiate nutritional from inflammatory effects limits the specificity of our findings and underscores the need for cautious interpretation of the mediation analyses. Fourth, the unique metabolic, inflammatory, and body composition profiles of individuals with SCI may alter the performance and interpretability of the CONUT score in ways that are not yet understood (52). Therefore, our findings should be considered exploratory and hypothesis-generating, and the CONUT score should be applied with caution in SCI populations until dedicated validation studies are completed. Fifth, the assessment of FI relied on self-reported questionnaires, which may be subject to recall bias or reporting bias due to social desirability, despite the use of validated instruments. Future studies should consider complementary assessment methods, such as prospective bowel diaries (e.g., 7-day or 14-day diaries) or clinician-administered interviews, to minimize these biases and improve the accuracy of FI ascertainment. Sixth, although comprehensive adjustments were made for potential confounders, residual confounding due to unmeasured variables may persist. For example, our study did not collect data on patients’ actual dietary intake, including fiber and fluid consumption (11, 53). These factors may indirectly and directly affect the frequency and severity of FI and may differ systematically between patients with better versus poorer nutritional status. Future prospective studies should include standardized dietary assessments (e.g., food frequency questionnaires) to clarify the independent role of nutritional status after accounting for these important confounders. Seventh, CONUT was calculated from single-time-point laboratory measurements, which may not reflect long-term nutritional status or fluctuations over time. Finally, the single-center design and exclusively Chinese population limit the generalizability of our findings to other ethnicities or geographic regions. External validation in multi-center, multi-ethnic, and international cohorts is essential before our findings can be considered generalizable to broader SCI populations.

## 5 Conclusion

CONUT was positively associated with both FI and FISI in a nearly linear dose–response manner and demonstrated moderate discriminative ability for FI, with superior performance compared with GNRI and PNI. In addition, IL-6 and UA partially accounted for these associations, suggesting inflammation and oxidative stress may be involved in this association. The robustness of the findings was further supported by consistent results across some subgroups and multiple sensitivity analyses.

## Data Availability

The original contributions presented in the study are included in the article/Supplementary material, further inquiries can be directed to the corresponding author.
