# Supplementary material for: Association of controlling nutritional status with fecal incontinence and fecal incontinence severity index in individuals with spinal cord injury: mediating roles of interleukin-6 and uric acid
Source: Front Nutr. 2026 Aug 17;13:1842981. doi: 10.3389/fnut.2026.1842981 (PMC13526543; doi:10.3389/fnut.2026.1842981)
Supplement: Supplementary file 1 [file Table_1.DOCX]

**Table S1.** Association between CONUt and FISI in patients with SCI stratified by various subgroups

|  | β (95%CI) | *P*-value | *P* for interaction |
| --- | --- | --- | --- |
| Age  < 60  ≥ 60 | 0.54 (0.47, 0.64)  0.46 (0.38, 0.57) | <0.001  <0.001 | 0.322 |
| Gender  Female  Male | 0.51 (0.43, 0.62)  0.53 (0.46, 0.64) | < 0.001  < 0.001 | 0.343 |
| Educational levels  Less than High-school  High school  College or above | 0.53 (0.47, 0.64)  0.55 (0.50, 0.67)  0.46 (0.41, 0.57) | < 0.001  < 0.001  < 0.001 | 0.415 |
| BMI  Underweight  Normal weight  Overweight  Obesity | 0.67 (0.53, 0.88)  0.40 (0.32, 0.53)  0.55 (0.46, 0.68)  0.61 (0.51, 0.73) | < 0.001  < 0.001  < 0.001  < 0.001 | 0.408 |
| Drinking status  Never  Former  Current | 0.45 (0.36, 0.59)  0.53 (0.43, 0.66)  0.64 (0.52, 0.78) | < 0.001  < 0.001  < 0.001 | 0.498 |
| Smoking status  Never  Former  Current | 0.50 (0.43, 0.63)  0.60 (0.52, 0.74)  0.68 (0,57, 0.83) | < 0.001  < 0.001  < 0.001 | 0.254 |
| Physical levels  Vigorous  Middle  low | 0.46 (0.37, 0.59)  0.58 (0.49, 0.64)  0.53 (0.45, 0.63) | < 0.001  < 0.001  < 0.001 | 0.356 |
| Hypertension  Yes  No | 0.62 (0.54, 0.74)  0.49 (0.43, 0.59) | <0.001  < 0.001 | 0.368 |
| Diabetes  Yes  No | 0.65 (0.56, 0.77)  0.51 (0.44, 0.64) | < 0.001  < 0.001 | 0.254 |
| Cardiovascular disease  Yes  No | 0.56 (0.48, 0.67)  0.48 (0.39, 0.60) | < 0.001  < 0.001 | 0.308 |

**Abbreviations:** BMI: body mass index, OR: odds ratio. Notes: All covariates were adjusted in the model, except for IL-6 and UA. *P* < 0.05 was regarded as statistically significant.

**Table S2.** The related indicator of the predictive effect of three malnutrition indices on FI risk in patients with SCI

|  | CONUT | GNRI | PNI |
| --- | --- | --- | --- |
| AUC (95%CI) | 0.788(0.759-0.818) | 0.632(0.603-0.662) | 0.626(0.595-0.656) |
| Sensitivity | 0.738 | 0.594 | 0.586 |
| Specificity | 0.701 | 0.585 | 0.576 |
| Accuracy | 0.719 | 0.613 | 0.594 |
| PPV | 0.732 | 0.620 | 0.605 |
| NPV | 0.694 | 0.604 | 0.592 |

**Abbreviations:** PNI: prognostic nutritional index, GNRI: geriatric nutritional risk index, CONUT: controlling nutritional status, AUC: area under the curve, PPV: positive predictive value, NPV: negative predictive value. **Notes:** the model included all covariates, except for UA and IL-6. *P* < 0.05 was regarded as statistically significant.

**Table S3.** Comparative analysis of three malnutrition indices

|  | CONUT vs PNI |  | CONUT vs GNRI |  | GNRI vs PNI |  |
| --- | --- | --- | --- | --- | --- | --- |
|  | Difference | *P-* value | Difference | *P*- value | Difference | *P*- value |
| FI  C-statistic (95%CI) | 0.162 (0.114, 0.223) | < 0.001 | 0.156 (0.108, 0.212) | < 0.001 | 0.008 (-0.003, 0.015) | 0.078 |

**Abbreviations:** PNI: prognostic nutritional index, GNRI: geriatric nutritional risk index. CONUT: controlling nutritional status. FI: fecal incontinence. **Notes:** The model included all covariates, except for UA and IL-6. *P* < 0.05 was regarded as statistically significant.

Table S4. Association of IL-6 and UA with FI and FISI in patients with SCI

|  | FI | | FISI | |  |
| --- | --- | --- | --- | --- | --- |
|  | OR (95%CI) | *P*-value | β (95%CI) | *P*-value | |
| IL-6 |  |  |  |  | |
| Continuous | 1.52 (1.41, 1.65) | < 0.001 | 0.65 (0.50, 0.81) | < 0.001 | |
| Q1 | Reference |  | Reference |  | |
| Q2 | 1.13 (1.05, 1.25) | 0.006 | 0.12 (0.04, 0.26) | 0.008 | |
| Q3 | 1.26 (1.14, 1.44) | < 0.001 | 0.41 (0.28, 0.62) | 0.001 | |
| Q4 | 1.64 (1.47, 1.78) | < 0.001 | 0.97 (0.68, 1.25) | < 0.001 | |
| *P* for trend | 0.001 |  | 0.001 |  | |
| UA |  |  |  |  | |
| Continuous | 1.65 (1.50, 1.89) | < 0.001 | 0.76 (0.64, 0.89) | < 0.001 | |
| Q1 | Reference |  | Reference |  | |
| Q2 | 1.30 (1.19, 1.49) | < 0.001 | 0.23 (0.12, 0.37) | < 0.001 | |
| Q3 | 1.53 (1.42, 1.70) | <0.001 | 0.52 (0.39, 0.71) | < 0.001 | |
| Q4 | 1.86 (1.65, 2.19) | <0.001 | 1.14 (0.92, 1.47) | < 0.001 | |
| *P* for trend | < 0.001 |  | < 0.001 |  | |

**Abbreviations:** UA: uric acid, IL-6: interleukin-6, FI: fecal Incontinence, FISI: fecal incontinence severity index. **Notes:** The model included all covariates, except for UA and IL-6. *P* < 0.05 was regarded as statistically significant.

**Table S5.** Association of IL-6 and UA with CONUT in patients with SCI

|  | FI | | FISI | |
| --- | --- | --- | --- | --- |
|  | β (95%CI) | *P*-value | β (95%CI) | *P*-value |
| FI |  |  | FISI |  |
| IL-6 Continuous | 0.54 (0.45, 0.67) | < 0.001 | 0.68 (0.55, 0.83) | < 0.001 |
| Q1 | Reference |  | Reference |  |
| Q2 | 0.26 (0.15, 0.38) | < 0.001 | 0.34 (0.22, 0.49) | < 0.001 |
| Q3 | 0.40 (0.31, 0.60) | < 0.001 | 0.52 (0.41, 0.68) | < 0.001 |
| Q4 | 0.73 (0.56, 0.88) | <0.001 | 0.83 (0.70, 0.99) | < 0.001 |
| *P* for trend | < 0.001 |  | < 0.001 |  |
| UA Continuous | 0.65 (0.55, 0.78) | < 0.001 | 0.79 (0.66, 0.93) | < 0.001 |
| Q1 | Reference |  | Reference |  |
| Q2 | 0.39 (0.25, 0.53) | < 0.001 | 0.42 (0.33, 0.57) | < 0.001 |
| Q3 | 0.51 (0.40, 0.66) | < 0.001 | 0.63 (0.52, 0.79) | < 0.001 |
| Q4 | 0.86 (0.74, 0.99) | < 0.001 | 0.91 (0.80, 1.13) | < 0.001 |
| *P* for trend | < 0.001 |  | < 0.001 |  |

**Abbreviations:** UA: uric acid, IL-6: interleukin-6, FI: fecal Incontinence, FISI: fecal incontinence severity index, Q1: quartile 1, Q2: quartile 2, Q3: quartile 3, Q4: quartile 4. **Notes:** The model included all covariates, except for UA and IL-6. *P* < 0.05 was regarded as statistically significant.

**Table S6.** Association of CONUT with FISI and FI in patients with SCI after imputation of missing data

|  | OR (95%CI) | *P*-value | β (95%CI) | *P*-value |
| --- | --- | --- | --- | --- |
| Model Ⅰ |  |  |  |  |
| Continuous | 1.38 (1.20, 1.64) | < 0.001 | 0.60 (0.42, 0.85) | < 0.001 |
| Q1 | Reference |  | Reference |  |
| Q2 | 1.03 (0.94, 1.20) | 0.221 | 0.06 (-0.10, 0.24) | 0.228 |
| Q3 | 1.31 (1.13, 1.58) | < 0.001 | 0.46 (0.23, 0.72) | < 0.001 |
| Q4 | 1.54 (1.40, 1.82) | < 0.001 | 0.67 (0.42, 0.92) | < 0.001 |
| *P* for trend | < 0.001 |  | 0.001 |  |
| Model Ⅱ |  |  |  |  |
| Continuous | 1.30 (1.14, 1.57) | < 0.001 | 0.48 (0.36, 0.76) | < 0.001 |
| Q1 | Reference |  | Reference |  |
| Q2 | 0.98 (0.89, 1.14) | 0.126 | 0.04 (-0.14, 0.25) | 0.207 |
| Q3 | 1.25 (1.09, 1.46) | < 0.001 | 0.37 (0.22, 0.64) | < 0.001 |
| Q4 | 1.46 (1.30, 1.69) | < 0.001 | 0.63 (0.38, 0.86) | < 0.001 |
| *P* for trend | 0.003 |  | 0.002 |  |
| Model Ⅲ |  |  |  |  |
| Continuous | 1.22 (1.07, 1.50) | 0.001 | 0.39 (0.27, 0.61) | < 0.001 |
| Q1 | Reference |  | Reference |  |
| Q2 | 0.92 (0.81, 1.05) | 0.213 | -0.03 (-0.27, 0.18) | 0.258 |
| Q3 | 1.12 (0.99, 1.28) | 0.056 | 0.17 (0.03, 0.38) | < 0.001 |
| Q4 | 1.40 (1.21, 1.65) | < 0.001 | 0.52 (0.30, 0.73) | < 0.001 |
| *P* for trend | 0.010 |  | 0.006 |  |

**Abbreviations:** Q1: quartile 1; Q2: quartile 2; Q3: quartile 3; Q4: quartile 4, OR: odds ratio. **Notes:** Model Ⅰ is a crude model. Model II adjusted for age, gender, educational levels. Model Ⅲ adjusted for all covariates, except for IL-6 and UA. *P* < 0.05 was regarded as statistically significant.

**Table S7.** Association of CONUT with FISI and FI in patients with SCI after excluding individuals with vascular, neoplastic, infectious, or gastrointestinal conditions.

|  | OR (95%CI) | *P*-value | β (95%CI) | *P*-value |
| --- | --- | --- | --- | --- |
| Model Ⅰ |  |  |  |  |
| Continuous | 1.43 (1.25, 1.68) | < 0.001 | 0.64 (0.46, 0.88) | < 0.001 |
| Q1 | Reference |  | Reference |  |
| Q2 | 1.06 (0.99, 1.23) | 0.226 | 0.10 (-0.02, 0.30) | 0.159 |
| Q3 | 1.34 (1.17, 1.62) | < 0.001 | 0.49 (0.27, 0.77) | < 0.001 |
| Q4 | 1.58 (1.43, 1.89) | < 0.001 | 0.78 (0.51, 0.98) | < 0.001 |
| *P* for trend | 0.001 |  | 0.001 |  |
| Model Ⅱ |  |  |  |  |
| Continuous | 1.34 (1.18, 1.60) | < 0.001 | 0.52 (0.38, 0.79) | < 0.001 |
| Q1 | Reference |  | Reference |  |
| Q2 | 1.01 (0.93, 1.18) | 0.158 | 0.03 (-0.07, 0.20) | 0.124 |
| Q3 | 1.30 (1.13, 1.49) | < 0.001 | 0.40 (0.26, 0.67) | < 0.001 |
| Q4 | 1.49 (1.34, 1.73) | < 0.001 | 0.66 (0.45, 0.93) | < 0.001 |
| *P* for trend | 0.003 |  | 0.002 |  |
| Model Ⅲ |  |  |  |  |
| Continuous | 1.26 (1.11, 1.53) | 0.001 | 0.45 (0.30, 0.68) | < 0.001 |
| Q1 | Reference |  | Reference |  |
| Q2 | 0.98 (0.85, 1.12) | 0.133 | -0.01 (-0.23, 0.20) | 0.217 |
| Q3 | 1.16 (1.04, 1.32) | 0.032 | 0.23 (0.06, 0.42) | 0.002 |
| Q4 | 1.43 (1.27, 1.68) | < 0.001 | 0.59 (0.40, 0.87) | < 0.001 |
| *P* for trend | 0.004 |  | 0.006 |  |

**Abbreviations:** Q1: quartile 1; Q2: quartile 2; Q3: quartile 3; Q4: quartile 4. OR: odds ratio. **Notes:** Model Ⅰ is a crude model. Model II adjusted for age, gender, educational levels. Model Ⅲ adjusted for all covariates, except for IL-6 and UA. *P* < 0.05 was regarded as statistically significant.

**Table S8.** Association of CONUT with FISI and FI in patients with SCI after additionally adding some potential confounding factors, such as certain medications (e.g., antibiotics and opioids)

|  | OR (95%CI) | *P*-value | β (95%CI) | *P*-value |
| --- | --- | --- | --- | --- |
| Model Ⅰ |  |  |  |  |
| Continuous | 1.33 (1.22, 1.58) | < 0.001 | 0.51 (0.37, 0.75) | < 0.001 |
| Q1 | Reference |  | Reference |  |
| Q2 | 1.01 (0.93, 1.20) | 0.156 | 0.02 (-0.08, 0.25) | 0.186 |
| Q3 | 1.28 (1.18, 1.45) | < 0.001 | 0.37 (0.22, 0.58) | < 0.001 |
| Q4 | 1.50 (1.36, 1.76) | < 0.001 | 0.63 (0.46, 0.87) | < 0.001 |
| *P* for trend | 0.001 |  | 0.003 |  |
| Model Ⅱ |  |  |  |  |
| Continuous | 1.26 (1.14, 1.53) | < 0.001 | 0.43 (0.30, 0.67) | < 0.001 |
| Q1 | Reference |  | Reference |  |
| Q2 | 0.97 (0.85, 1.10) | 0.179 | -0.05 (-0.20, 0.13) | 0.216 |
| Q3 | 1.20 (1.10, 1.35) | < 0.001 | 0.32 (0.16, 0.53) | < 0.001 |
| Q4 | 1.40 (1.25, 1.64) | < 0.001 | 0.55 (0.38, 0.76) | < 0.001 |
| *P* for trend | 0.003 |  | 0.006 |  |
| Model Ⅲ |  |  |  |  |
| Continuous | 1.18 (1.10, 1.48) | 0.001 | 0.36 (0.26, 0.58) | < 0.001 |
| Q1 | Reference |  | Reference |  |
| Q2 | 0.93 (0.81, 1.06) | 0.205 | -0.12 (-0.27, 0.04) | 0.268 |
| Q3 | 1.13 (1.02, 1.29) | 0.047 | 0.14 (0.03, 0.34) | 0.002 |
| Q4 | 1.37 (1.21, 1.54) | < 0.001 | 0.48 (0.29, 0.70) | < 0.001 |
| *P* for trend | 0.005 |  | 0.008 |  |

**Abbreviations:** Q1: quartile 1; Q2: quartile 2; Q3: quartile 3; Q4: quartile 4. OR: odds ratio. **Notes:** Model Ⅰ is a crude model. Model II adjusted for age, gender, educational levels Model Ⅲ adjusted for all covariates, except for UA and IL-6. *P* < 0.05 was regarded as statistically significant.

**Table S9.** Association of CONUT with FISI and FI in patients with SCI after additionally adding some inflammatory factors, such as CRP, TNF‑α, and IL‑1β.

|  | OR (95%CI) | *P*-value | β (95%CI) | *P*-value |
| --- | --- | --- | --- | --- |
| Model Ⅰ |  |  |  |  |
| Continuous | 1.30 (1.18, 1.51) | < 0.001 | 0.47 (0.31, 0.76) | < 0.001 |
| Q1 | Reference |  | Reference |  |
| Q2 | 0.98 (0.89, 1.15) | 0.253 | -0.02 (-0.09, 0.14) | 0.213 |
| Q3 | 1.20 (1.12, 1.41) | < 0.001 | 0.32 (0.21, 0.53) | < 0.001 |
| Q4 | 1.42 (1.32, 1.68) | < 0.001 | 0.56 (0.44, 0.75) | < 0.001 |
| *P* for trend | 0.003 |  | 0.005 |  |
| Model Ⅱ |  |  |  |  |
| Continuous | 1.23 (1.11, 1.45) | < 0.001 | 0.37 (0.24, 0.55) | < 0.001 |
| Q1 | Reference |  | Reference |  |
| Q2 | 0.93 (0.82, 1.08) | 0.241 | -0.06 (-0.17, 0.11) | 0.255 |
| Q3 | 1.13 (1.03, 1.29) | < 0.001 | 0.21 (0.06, 0.45) | < 0.001 |
| Q4 | 1.34 (1.21, 1.52) | < 0.001 | 0.46 (0.31, 0.68) | < 0.001 |
| *P* for trend | 0.005 |  | 0.009 |  |
| Model Ⅲ |  |  |  |  |
| Continuous | 1.14 (1.05, 1.32) | 0.003 | 0.30 (0.17, 0.49) | < 0.001 |
| Q1 | Reference |  | Reference |  |
| Q2 | 0.90 (0.79, 1.04) | 0.272 | -0.16 (-0.29, 0.06) | 0.291 |
| Q3 | 1.09 (1.01, 1.23) | 0.043 | 0.12 (0.02, 0.28) | 0.035 |
| Q4 | 1.31 (1.16, 1.46) | < 0.001 | 0.41 (0.23, 0.65) | < 0.001 |
| *P* for trend | 0.008 |  | 0.014 |  |

**Abbreviations:** Q1: quartile 1; Q2: quartile 2; Q3: quartile 3; Q4: quartile 4. OR: odds ratio. **Notes:** Model Ⅰ is a crude model. Model II adjusted for age, gender, educational levels Model Ⅲ adjusted for all covariates, except for UA and IL-6. *P* < 0.05 was regarded as statistically significant.

**Table S10.** Association of CONUT with FISI and FI in patients with SCI after additionally adjusting for some specific bowel management strategies, including laxative use, enema use, digital evacuation, antidiarrheal medications, and structured bowel management programs.

|  | OR (95%CI) | *P*-value | β (95%CI) | *P*-value |
| --- | --- | --- | --- | --- |
| Model Ⅰ |  |  |  |  |
| Continuous | 1.25 (1.15, 1.46) | < 0.001 | 0.40 (0.27, 0.62) | < 0.001 |
| Q1 | Reference |  | Reference |  |
| Q2 | 0.95 (0.86, 1.13) | 0.242 | -0.07 (-0.17, 0.21) | 0.287 |
| Q3 | 1.18 (1.07, 1.34) | < 0.001 | 0.25 (0.14, 0.46) | < 0.001 |
| Q4 | 1.37 (1.28, 1.62) | < 0.001 | 0.51 (0.36, 0.67) | < 0.001 |
| *P* for trend | 0.005 |  | 0.009 |  |
| Model Ⅱ |  |  |  |  |
| Continuous | 1.20 (1.10, 1.38) | < 0.001 | 0.31 (0.18, 0.49) | < 0.001 |
| Q1 | Reference |  | Reference |  |
| Q2 | 0.91 (0.81, 1.04) | 0.213 | -0.11 (-0.21, 0.07) | 0.292 |
| Q3 | 1.12 (1.01, 1.24) | < 0.001 | 0.18 (0.02, 0.36) | < 0.001 |
| Q4 | 1.31 (1.20, 1.51) | < 0.001 | 0.40 (0.24, 0.56) | < 0.001 |
| *P* for trend | 0.009 |  | 0.013 |  |
| Model Ⅲ |  |  |  |  |
| Continuous | 1.12 (1.04, 1.26) | 0.011 | 0.25 (0.12, 0.37) | < 0.001 |
| Q1 | Reference |  | Reference |  |
| Q2 | 0.89 (0.79, 1.01) | 0.353 | -0.18 (-0.32, 0.03) | 0.412 |
| Q3 | 1.06 (0.96, 1.18) | 0.089 | 0.09 (-0.08, 0.20) | 0.076 |
| Q4 | 1.22 (1.13, 1.41) | < 0.001 | 0.36 (0.22, 0.51) | < 0.001 |
| *P* for trend | 0.012 |  | 0.017 |  |

**Abbreviations:** Q1: quartile 1; Q2: quartile 2; Q3: quartile 3; Q4: quartile 4. OR: odds ratio. **Notes:** Model Ⅰ is a crude model. Model II adjusted for age, gender, educational levels Model Ⅲ adjusted for all covariates, except for UA and IL-6. *P* < 0.05 was regarded as statistically significant.
